# Tellurium Nanowires for Lithium‐Metal Anode Stabilization in High‐Performance Anode‐Free Li–S Batteries

**DOI:** 10.1002/smsc.202300088

**Published:** 2023-08-22

**Authors:** Hyunki Sul, Jiarui He, Arumugam Manthiram

**Affiliations:** ^1^ Walker Department of Mechanical Engineering The University of Texas at Austin Austin TX 78712 USA

**Keywords:** anode-free cells, lithium-metal stabilization, lithium–sulfur batteries, separator coating, tellurium nanowires

## Abstract

Enhancing the reversibility of Li is crucial for extending the cycle life of Li‐limited anode‐free lithium–sulfur (Li–S) batteries. Incorporating tellurium (Te) in the system has proven to be highly effective by its reaction with polysulfides and forming a passivating interfacial layer on Li surface, which reduces the Li‐ion diffusion barrier. However, due to the poor utilization of Te, a significant amount of Te is required to improve cell cycling performance. To address this, nanowire‐structured Te (TeNW) is synthesized via a hydrothermal method and applied to Li_2_S‐based anode‐free cells to minimize the Te content in the system while extending the cell cycle life. Coating TeNW onto the separator greatly enhances Te utilization and demonstrates a significant cycle life improvement (38% retention over 300 cycles) with only 4 wt% TeNW content relative to the active material. The versatility of TeNW is further demonstrated by utilizing them with carbon nanotubes as the anode substrate. The exceptional performance of TeNW is attributed to the high‐surface‐area nanostructure and excellent conductive network, facilitating efficient electron transfer during cell cycling. These advantageous properties position TeNW as a promising material to enhance the cycle life of Li‐limited Li–S batteries.

## Graphical abstract

High‐surface‐area nanostructured tellurium nanowires utilized in lithium‐limited lithium–sulfur full cells enhance the cycling performance by stabilizing the lithium‐metal anode. Coating the nanowires on the separator or using them as an anode substrate maximizes tellurium utilization, resulting in improved cycle life with minimal tellurium content.

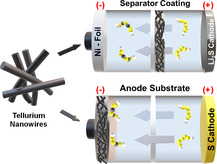

## 1 Introduction

The exploration of new battery chemistries beyond lithium‐ion batteries (LIBs) is imperative and essential to meet the growing market demands.^[^ ^1^, ^2^, ^3^, ^4^, ^5^ ^]^ The limitations of current LIBs stem from the relatively low specific capacity (<220 mAh g^−1^) and the heavy weight of conventional transition‐metal oxide cathodes. These factors restrict the specific energy density of LIBs within 250 Wh kg^−1^.^[^ ^6^, ^7^, ^8^ ^]^ Meanwhile, lithium–sulfur (Li–S) batteries offer a high theoretical specific capacity of 1675 mAh g^−1^ and can achieve a full‐cell‐level theoretical energy density of >600 Wh kg^−1^.^[^ ^9^, ^10^ ^]^ Along with its high energy density, environmental friendliness, and cost‐effectiveness, Li–S battery technology has garnered significant attention as a promising candidate for next‐generation energy storage systems.^[^ ^11^, ^12^, ^13^ ^]^

In the Li–S system, the high theoretical capacity of sulfur necessitates the inclusion of a corresponding quantity of Li. Additionally, to compensate for Li loss caused by its reaction with electrolyte and formation of solid‐electrolyte interphase (SEI) during cycling, an excess amount of Li is typically used in the system.^[^ ^14^, ^15^, ^16^ ^]^ This excess Li is incorporated into the cell in the form of a thick Li‐metal foil (with a thickness exceeding 500 μm), resulting in a negative‐to‐positive capacity (N/P) ratio greater than 20. However, such a high N/P ratio leads to an increase in excess Li weight, penalizing the specific energy of the Li–S battery to limit it to around 150 Wh kg^−1^.^[^ ^17^ ^]^ Moreover, considering the rising price of Li, the inclusion of large quantities of Li in the cell undermines the cost‐effectiveness advantage associated with Li–S batteries.^[^ ^18^, ^19^ ^]^ Consequently, there is a growing focus on approaches that aim to decrease the N/P ratio to achieve high energy density and low‐price Li–S batteries.^[^ ^20^, ^21^, ^22^, ^23^ ^]^

Among the various approaches to reduce the N/P ratio in Li–S batteries, anode‐free full cells based on Li_2_S cathodes have emerged as a promising avenue.^[^ ^24^, ^25^, ^26^, ^27^ ^]^ By combining a fully lithiated cathode with a bare current collector on the anode, it is possible to maintain the N/P ratio precisely equal to 1.^[^ ^28^ ^]^ Given the energy‐, process‐, and cost‐intensive nature of manufacturing thin Li foils, the use of prelithiated Li_2_S‐cathode‐based anode‐free cells presents notable advantages. First, they enable the maximum achievable energy density to be delivered without the presence of excess Li. Second, anode‐free cells provide the flexibility to utilize various types of anodes, including carbon‐based, silicon‐based, and metal‐oxide‐based materials to improve anode stability. Finally, anode‐free cells are free from self‐discharging issues, as Li_2_S cathodes start in a fully discharged state. These advantages make anode‐free cells with prelithiated Li_2_S cathodes a promising direction for research and development in advanced battery systems.

Nevertheless, the absence of excess Li in anode‐free cells poses a challenge in terms of achieving extended cell cycle life. Numerous investigations have highlighted the prominent role of Li‐metal anode degradation in determining the long‐term cycling performance, particularly in Li‐limited anode‐free cell configurations.^[^ ^29^, ^30^, ^31^, ^32^ ^]^ Consequently, addressing the degradation issues associated with Li‐metal anode is essential to optimize the performance and durability of anode‐free cell systems. Several approaches have been attempted to improve the stability of the Li‐metal anode, including the use of 3D hosts to enhance Li nucleation,^[^ ^33^, ^34^, ^35^, ^36^ ^]^ pretreatments of the Li surface to minimize side reactions,^[^ ^37^, ^38^, ^39^, ^40^ ^]^ and in situ SEI modifications.^[^ ^41^, ^42^, ^43^, ^44^ ^]^ These strategies aim to mitigate the degradation mechanisms occurring at the Li‐metal interface and improve the overall stability and cyclability of anode‐free cells.

One effective approach to enhance the reversibility of the Li‐metal anode is through the formation of a highly Li‐ion conductive SEI layer. Instead of the conventional Li_2_S‐rich SEI, formation of ternary sulfide‐rich SEI, such as Li_3_PS_4_, Li_2_SnS_3_, etc., has shown improved performance in terms of Li stripping and plating, as well as stability against polysulfides.^[^ ^45^, ^46^, ^47^, ^48^ ^]^ Especially, the inclusion of tellurium (Te) as the cathode additive has been found to significantly enhance the cycling performance of anode‐free cells by spontaneously reacting with polysulfides and forming a lithium polytellurosulfide (Li_2_Te_*x*_S_*y*_)‐rich SEI.^[^ ^49^ ^]^ As Te being a group 16 element, it shares similar chemistry with sulfur, which makes it readily form Li_2_Te_*x*_S_*y*_ compounds.^[^ ^50^ ^]^ Additionally, Te has the ability to form soft Lewis acid cations (Te^4+^), which have a preference for soft Lewis bases such as S^2−^ sulfides, favoring the formation of Li_2_Te_*x*_S_*y*_ at the Li‐metal surface.^[^ ^51^, ^52^ ^]^ The presence of a superior Li‐ion conductive SEI layer enables homogenous and dense Li morphology, which mitigates parasitic side reactions at the Li surface. Consequently, the Li inventory is well maintained throughout cell cycling.

However, in these studies, the use of commercial Te as a Li_2_S cathode additive demonstrated cycle life improvement with an addition of approximately 20 wt% Te. As widely known, Te is a heavy transition metal (127.6 g mol^−1^) and an expensive material ($86.1 per kg).^[^ ^53^ ^]^ Such an excess amount of Te addition in the Li–S system significantly diminishes the advantages of Li–S batteries in terms of gravimetric energy density and cost‐effectiveness. Therefore, finding the optimal balance where Te content is minimized while effectively stabilizing the Li‐metal anode is critical from a commercial viability standpoint. Ideally, by reducing Te content to less than 5 wt% relative to Li_2_S, the mass contribution of Te in the entire cell under practical pouch cells can be minimized to a mere 0.58%, making it the lowest among all cell components (Table S1, Supporting Information).

## 2 Results and Discussion

In this regard, what is the minimum Te content that can be reduced while still achieving significant cycling retention improvement in anode‐free cells? To evaluate the impact of varying Te content at the cathode on cycling performance, commercial Te powder was mechanically mixed with Li_2_S and carbon in different weight percentages; 10, 5, and 0 wt%, and subsequently applied as a cathode material. Due to the ability of Te to stabilize the Li‐metal anode, a Li‐limited anode‐free cell configuration was employed to effectively compare the impact of varying Te content in the system. The Li_2_S loading was 3 mg cm^−2^, and 45 μL of electrolyte was injected. As Li_2_S was used as a cathode material, specific capacities are calculated on Li_2_S basis.

**Figure 1:**
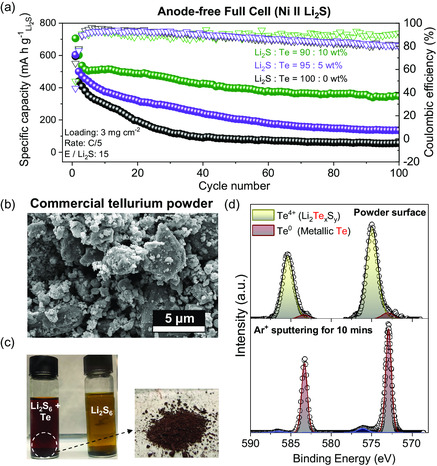
a) Long‐term cycling performance of anode‐free Ni || Li_2_S full cells with 10, 5, and 0 wt% Te applied at the cathode compared to Li_2_S. b) SEM image of commercial Te powder. c) Color change of Li_2_S_6_ when reacted with commercial Te powder and a digital image of dried Li_2_S_6_ + Te. d) Te 3d XPS spectra of dried Li_2_S_6_ + Te at the surface and after 10 min of Ar^+^ sputtering.

As shown in **Figure** **1**a, the conventional anode‐free cell without Te shows a rapid capacity decay within 30 cycles, leading to a poor capacity retention of 11% at the end of 100 cycles. When 5 wt% of Te powder was added to the cathode, a slight cycle life improvement is detected (22% retention over 100 cycles). To further enhance the cyclability of the cell, 10 wt% of Te powder was applied to the cathode, resulting in a significant cycle life improvement (50% retention over 100 cycles). Also, the increase in initial discharge capacity is detected, which might be coming from the catalytic effect of Te on sulfur redox.^[^ ^54^, ^55^, ^56^, ^57^ ^]^ Thus, it is apparent that achieving a meaningful improvement in anode‐free cell performance necessitates a minimum of 10 wt% of Te in the Li_2_S cathode.

To investigate the origin of why a significant amount of Te is required, the reaction between commercial Te powder and lithium polysulfide was studied. As widely known, the mechanism of the Te stabilizing the Li‐metal anode involves its reaction with lithium polysulfide, resulting in the formation of a highly Li‐ion conductive Li_2_Te_*x*_S_*y*_ layer at the surface of Li metal.^[^ ^49^, ^50^ ^]^ Scanning electron microscopy (SEM) image confirms that the commercial Te powder has a secondary particle size of up to ≈5 μm (Figure 1b). When 0.01 m of the micrometer‐sized Te powder got dispersed in 0.05 m Li_2_S_6_ solution in 1,3‐dioxolane and 1,2‐dimethoxyethane (DOL/DME), the change in color from yellow to red is detected. After drying out the supernatant, dark‐red colored powder was collected, as shown in Figure 3c. This dark‐red powder, which is known to be the color of polytellurosulfide (Li_2_Te_*x*_S_*y*_) species, shows a spontaneous reaction between Te and Li_2_S_6_. X‐ray photoelectron spectroscopy (XPS) analysis of the dried polytellurosulfide powder showed that Te is present as Te^4+^ at the surface of the powder (574.9 eV), which confirms the redox reaction between Te and Li_2_S_6_ (Figure 1d). The oxidation of Te from Te^0^ to Te^4+^ is balanced by the reduction of S^0^ to S^1−^ and S^2−^, as shown in Figure S1, Supporting Information. To analyze the bulk of the powder, Ar^+^ sputtering of the surface was conducted for 10 min. Surprisingly, the resulting spectrum shows the disappearance of the reduced Te^4+^ peak and the emergence of metallic Te^0^ peak at 572.9 eV. It is clearly shown that the reaction between Te and Li_2_S_6_ to form the Li_2_Te_*x*_S_*y*_ only happened at the surface, and the bulk still remains as metallic Te^0^.

A similar phenomenon is expected to occur inside the Li–S batteries. Upon cell cycling, micrometer‐sized Te powder might mainly react with lithium polysulfides only at the surface, resulting in poor utilization of Te. The insulating nature of sulfur or Li_2_S could exacerbate the low utilization of Te. In the end, an excess amount of Te is required to achieve cycling retention improvement. As mentioned earlier, the inclusion of a high content of Te within the system would offset the advantages of Li–S batteries, as Te is a heavy and costly metal. Therefore, to overcome the poor Te utilization, it is necessary to use the high‐surface‐area structured Te to maximize its reaction with the polysulfides.

Typically, nanoscale materials exhibit a substantially higher surface‐area‐to‐volume ratio compared to their bulk counterparts, which results in shorter diffusion distances for electrons and Li ions within the nanoparticles.^[^ ^57^, ^58^, ^59^ ^]^ In particular, nanowires are well known for their superior mechanical and thermal stability, as well as their excellent electrical conductivity.^[^ ^60^, ^61^, ^62^ ^]^ It has been reported that the high‐surface area nanowire structured material can be synthesized through the hydrothermal reaction.^[^ ^63^, ^64^, ^65^ ^]^ Building upon this rationale, attempts have been made to synthesize Te materials in the form of nanowires, capitalizing on their unique structure.

**Figure 2:**
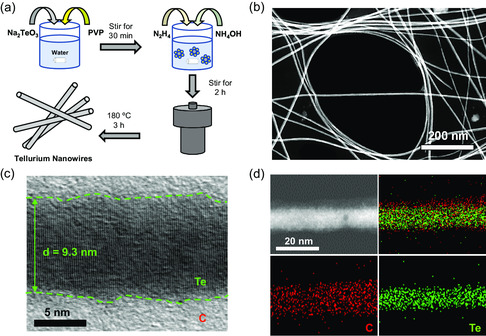
a) Synthesis schematic of TeNW. b) Low‐magnification and c) high‐magnification TEM images of TeNW. d) EDX elemental mapping of TeNW.

Figure 1a illustrates the addition of Na_2_TeO_3_ as a Te source in deionized water, with poly(vinylpyrrolidone) (PVP) acting as a surfactant. After the solution was mixed for 30 min, hydrazine monohydrate and aqueous ammonia solution were introduced. The purpose of hydrazine is to mineralize and nanocrystalize the Te ions during the hydrothermal synthesis,^[^ ^66^, ^67^ ^]^ and ammonia contributes to preventing large Te crystal growth and improving the uniformity of the nanowire‐structured Te.^[^ ^68^ ^]^ Subsequently, the closed reaction vessel was subjected to a temperature ramp up to 180 °C, creating high‐temperature and high‐pressure conditions to facilitate the hydrothermal process, which yields nanowire‐structured Te (TeNW). The elongated and rod‐like nanowire structure was visualized with transmission electron microscopy (TEM), as shown in **Figure** **2**b. The high‐magnification TEM image shows that the diameter of the nanowire is ≈9 nm (Figure 2c). A well‐resolved lattice fringes corresponds to the crystalline planes of hexagonal Te, whereas the amorphous region indicates the thin carbon coating at the surface. The source of carbon is coming from the reaction leftover from the PVP. The energy‐dispersive X‐ray spectroscopy (EDX)–elemental mapping confirms the core of the nanowire is Te (green), whereas a surface coating consists of carbon (red), as shown in Figure 2d.

The amount of carbon coating on TeNW was quantified by thermogravimetric analysis (TGA) under N_2_ atmosphere (Figure S2, Supporting Information). In the temperature range of 450–520 ºC, weight loss is observed, which is attributed to the evaporation of Te. The content of carbon coating in the synthesized TeNW is found to be 10 wt%. The crystalline structure of TeNW was further characterized with X‐ray diffraction (XRD). As illustrated in Figure S3, Supporting Information, TeNW exhibits distinct peaks corresponding to the hexagonal Te crystal structure, which aligns well with the standard data (JCPDS 36‐1452). The series of material characterization reveals that the thin carbon‐coated TeNW was successfully synthesized through the hydrothermal reaction.

To evaluate the potential application of TeNW in an anode‐free Li–S system, 5 wt% of TeNW was mechanically mixed with Li_2_S and applied to the cathode. As shown in Figure S4, Supporting Information, the cell with TeNW exhibits an initial discharge capacity of 669 mAh g^−1^ with 29% capacity retention at the end of 100 cycles, whereas the cell with the same wt% of Te powder shows an initial capacity of 604 mAh g^−1^ with 22% retention. Contrary to expectations, despite its high‐surface‐area nanostructure, the cycling performance is not significantly improved with TeNW compared to the same wt% of Te powder. It is assumed that even with an increased surface area of nanostructured Te, the insulating characteristics of Li_2_S play a dominant role in impeding the utilization of TeNW. Note that the ultimate goal is to discover a strategy to enhance cell cycling performance in the Li–S system while minimizing the Te content. Therefore, adding TeNW into a cathode composed of Li_2_S with poor ionic and electronic conductivity may not be an effective strategy.

**Figure 3:**
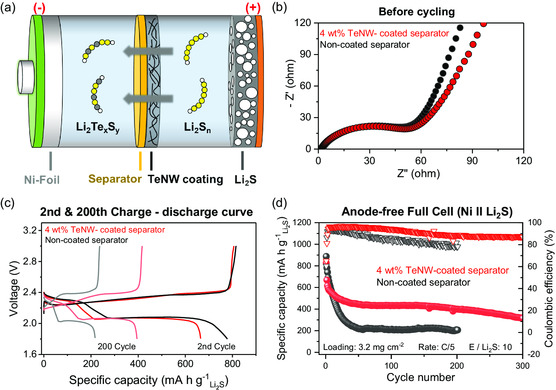
a) Schematic of the TeNW‐coated separator in anode‐free cell configuration. b) Nyquist plots before cycling, c) 2nd and 200th cycle charge–discharge curves, and d) long‐term cycling performance of 4 wt% TeNW‐coated separator and uncoated separator cells.

An alternative approach to incorporate TeNW into the Li–S system is by coating it onto the separator. Separator coating offers the advantage of isolating TeNW from the insulating Li_2_S and allows for an easy application of the existing slurry coating method from a manufacturing standpoint. It is expected that during cell cycling, polysulfides dissolved in the electrolyte can spontaneously react with TeNW at the separator and migrate to Li‐metal anode to form a favorable SEI layer (**Figure** **3**a). TeNW was homogenously mixed with commercial Soteras binder (95:5 wt%) in water and subsequently blade‐casted onto Celgard 2500 separator. The TeNW has a nanosized diameter and a rod‐like structure, which enables a uniform and thin coating in a wide area of the separator. SEM image shows a homogenous distribution of the TeNW coating at the surface of the separator (Figure S5, Supporting Information) and the thickness of the coating is ≈7.3 μm (Figure S6, Supporting Information).

The TeNW‐coated separator was combined with Li_2_S cathode in an anode‐free cell configuration to evaluate the electrochemical performance. Li_2_S loading was 3.2 mg cm^−2^ and the total mass of TeNW that were coated onto the separator was 0.13 mg cm^−2^. The content of TeNW was purposely controlled to a low percentage of 4 wt% compared to Li_2_S in the cell. To measure the increase in cell resistance that may occur due to the separator coating, electrochemical impedance spectroscopy (EIS) was conducted. Despite the addition of a new layer consisting of TeNW to the separator, the charge‐transfer resistance of the pristine cells does not show a significant difference compared to the uncoated case, as shown in Figure 3b. This result is also observed in the charge–discharge curves of the second cycle. Based on Figure 3c, the cell with the TeNW coating shows a similar voltage polarization as the cell without Te coating. The results from the EIS and voltage curve analysis suggest that the ultrathin TeNW layer at the separator does not significantly increase the cell resistance or hinder the reaction kinetics.

To compare the cell performance based on the separator coating side, two configurations were examined: one with the coating side facing the cathode and the other with the coating side facing the anode, as depicted in Figure S7a, Supporting Information. From the cycling performance shown in Figure S7b, Supporting Information, the TeNW‐coated side facing the cathode shows slightly better capacity retention and higher average Coulombic efficiency (67% retention, 93.0% Coulombic efficiency) compared to the cell with the TeNW‐coated side facing the anode (63% retention, 91.9% Coulombic efficiency) throughout 30 cycles. The exposure of TeNW‐coated side toward the cathode might have maximized the reaction between TeNW and polysulfides to form polytellurosulfide species, which facilitates the generation of stable SEI on Li surface. Moving forward, all cells were assembled with the TeNW‐coated side facing the cathode, considering the superior cycling performance observed in this configuration.

The enhanced cycling stability attributed to the TeNW was confirmed through a long‐term cycling performance measurement conducted at C/10 rate activation for three cycles, followed by C/5 rate. Figure 3d shows that regardless of a low TeNW content of 4 wt% in the system, the anode‐free cell delivers good cycle life improvement at the end of 300 cycles (316 mAh g^−1^, 38% retention), exhibiting a small capacity decay of 1.7 mAh g^−1^ per cycle with an average Coulombic efficiency of 91.3%. The initial capacity fade during the first five cycles is attributed to the consumption of S and Li during the formation of a robust SEI, after which the cell exhibits excellent capacity retention. The gradual attenuation of Te signal on the coated separator, as shown in Figure S8, Supporting Information, reveals the consumption of Te by reaction with the polysulfides to form a stable SEI. In contrast, the control cell without the Te in the system shows a rapid capacity decay, losing almost 75% of its capacity within the first 50 cycles and exhibiting a lower average Coulombic efficiency of 85.3%. Thus, it is shown that the application of TeNW on the separator is an effective way to significantly improve cell cycling performance while reducing Te content down to 4 wt%.

One important aspect to consider is whether the improvement in cell cycling performance is attributed to TeNW itself or by the additional separator coating. As widely known, the addition of a layer on the separator might have some degree of suppression of shuttle effect by physically anchoring the polysulfides, which can improve the cycling performance of anode‐free cells. Even though the TeNW on the separator seems to disappear during cycling, the binder used to coat the separator may remain in the separator and potentially have an impact on suppressing polysulfide shuttling. To investigate whether the binder itself has any polysulfide‐shuttle‐suppressing effect, a shuttle current test was conducted where only the binder was coated onto the separator without the addition of TeNW. As shown in Figure S9, Supporting Information, both the coated separator and the uncoated separator cell show a similar shuttle current of ≈0.11 mA, which indicates that the binder itself used on the separator coating has negligible impact on the suppression of polysulfide migration. It is confirmed that the dominant mechanism for improving the cell cycling retention is not by the separator coating itself, but by the TeNW.

For a direct comparison with TeNW, commercial Te powder was also coated on the separator using the same binder. The uneven particle size distribution of Te powder hampers the homogenous mixing with the binder. Thus, carbon was needed to enable the Te powder coating onto the separator. As shown in Figure S10a, Supporting Information, the separator coated with Te powder exhibits irregular Te and carbon distribution. Additionally, the commercial Te powder has a large particle size, which limits the minimum achievable coating thickness to approximately 12 μm (Figure S10b, Supporting Information). Due to the substantial coating thickness, when combined with a 3.2 mg cm^−2^ loading of a Li_2_S cathode, the total Te content turned out to be 15 wt% relative to Li_2_S. It is well known that such irregularity in the separator coating can lead to an inhomogeneous Li‐ion flux, resulting in nonuniform Li stripping and plating at the anode. Furthermore, thicker separator coating causes an ohmic drop in the cell, which causes increased voltage polarization and larger charge‐transfer resistance, as shown in Figure S11a,b, Supporting Information. Although the Te powder‐coated separator cells contained a high Te content of 15 wt%, the cells did not exhibit a dramatic improvement in cycling stability compared to the control cell (Figure S12, Supporting Information). Based on these results, it can be concluded that using TeNW instead of Te powder is a better choice to apply it to the separator and achieve the most efficient cycling performance enhancement while minimizing the Te content.

**Figure 4:**
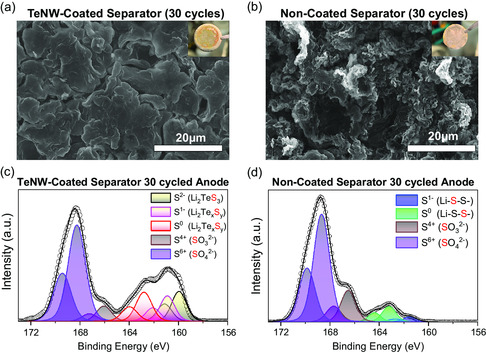
a,b) SEM images of Li‐metal anode surfaces after 30 cycles: a) TeNW‐coated separator cell and b) uncoated separator cell. The insets are the digital images of Li‐plated Ni foils from the anode‐free cells after 30 cycles. c,d) S 2p XPS spectra of Li‐metal surface after 30 cycles: c) TeNW‐coated separator cell and d) uncoated separator cell.

In order to understand the effects of the TeNW‐coated separator on the anode side, the deposited Li in anode‐free full cells after 30 cycles were analyzed. SEM image shows that Li cycled with TeNW‐coated separator reveals a dense, planar, and smooth morphology (**Figure** **4**a). The red‐colored areas generated on Li‐metal surface (Figure 4a inset image) indicate the formation of a SEI layer containing lithium polytellurosulfides. In contrast, Li with conventional uncoated separator shows mossy and filamentous deposition (Figure 4b). The contrasting Li morphologies help explain the differences in capacity retention observed in Figure 3d. The mossy deposition of Li leads to severe parasitic side reaction between Li with electrolyte to form a “dead” metallic Li. In contrast, the dense and uniform morphology of Li minimizes the reaction sites to preclude such an irreversible loss of Li inventory during cycling.

To determine the underlying cause of the contrasting Li morphologies, the chemical composition of the SEI layer after 30 cycles was analyzed with XPS. Figure 4c presents the S 2p spectra of the Li surface for the cell with the TeNW‐coated separator. It is shown that reduced sulfide peaks (S^2−^) are detected at 159.9 eV, indicating the formation of Li_2_TeS_3_ at the surface of Li‐metal anode.^[^ ^49^ ^]^ Additionally, the peaks of terminal sulfur (S^1−^) and bridging sulfur (S^0^) at 160.9 and 162.8 eV, respectively, suggest the presence of intermediate polytellurosulfide species. It has been reported that the Te containing ternary sulfide (Li_2_Te_*x*_S_*y*_) species at the SEI acts as a good Li‐ion conductor to efficiently distribute the Li‐ion flux, leading to a homogenous Li plating and stripping.^[^ ^51^, ^69^ ^]^

The formation of reaction products between Te and polysulfides can also be confirmed from the Te 3d XPS spectra. Figure S13, Supporting Information, shows the presence of Te^4+^ peaks at 574.8 eV on the surface of the SEI, which results from the formation of Li_2_TeS_3_. Tellurium oxide peaks at 575.5 eV might be coming from the reaction between Te and the electrolyte. To confirm the nature of the SEI layer containing Te, Ar^+^ sputtering was conducted for 10 min. As it gets closer to the bulk deposited Li, the strongly reducing nature of Li metal leads to a further reduction in Te into Te^1−^ (Li_2_Te_2_) or Te^2−^ (Li_2_Te), which are shown in the peaks at 571.7 and 569.9 eV, respectively. The transition from Li_2_TeS_3_ to Li_2_Te_2_ and Li_2_Te, as getting closer to bulk Li metal, shows a similar trend as in the literatures.^[^ ^49^, ^50^ ^]^ XPS spectra clearly reveal that the TeNW from the separator undergoes the reaction with polysulfides and migrates to the Li‐metal anode to form a good Li‐ion conducting SEI layer.

**Figure 5:**
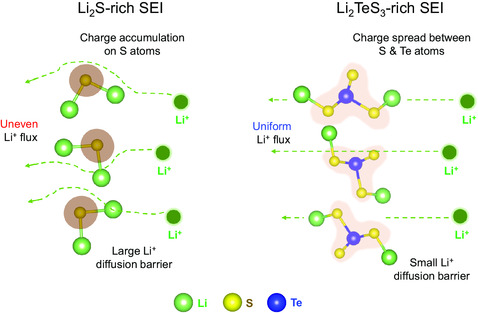
Schematic of the Li‐ion diffusion trend in conventional Li_2_S‐rich SEI and Li_2_TeS_3_‐rich SEI.

In contrast, the cycled cell with a conventional uncoated separator shows a distinctive S 2p spectrum. As shown in Figure 4d, the peaks of terminal sulfur S^1−^ at 161.4 eV and bridging sulfur S^0^ at 163.16 eV are known to be coming from the lithium polysulfides. The dominating intensity of oxidized sulfur species (S^4+^, S^6+^) indicates that severe electrolyte decomposition is taking place at Li surface. Such a difference in SEI chemical composition and resulting Li‐metal morphological changes observed in this study are consistent with previous reports. Compared to the conventional binary sulfide (Li_2_S)‐rich SEI, the ternary sulfide (Li_2_Te_*x*_S_*y*_)‐rich SEI has a highly covalent characteristic due to the small electronegativity difference between S (2.58) and Te (2.10). As a result, the ternary sulfide (Li_2_Te_*x*_S_*y*_)‐dominant SEI leads to a low Li‐ion diffusion barrier, allowing for smoother Li‐ion transport. Consequently, Li‐ion flux becomes more uniform, which facilitates a homogenous Li plating and stripping processes, as demonstrated in **Figure** **5**. Therefore, coating the separator with TeNW can provide analogous stabilizing effect on the Li‐metal anode as the previously reported mechanisms with a much lower TeNW content of only 4 wt% in the system.

To further demonstrate the universal applicability of TeNW in Li–S system, the possibility of applying TeNW to other cell components was investigated. As the mechanism by which TeNW enhances cell cycle life mainly occurs at the Li‐metal anode, direct application of TeNW to the anode can be an effective approach. Based on this motivation, a method of using TeNW with carbon nanotube (CNT) as an anode substrate was attempted. The TeNW and CNT in a 1:9 weight ratio were dispersed in ethanol/water (1:1 vol%) solution via probe‐sonication and subsequently vacuum filtered to form a binder‐free TeNW @ CNT sheet (Figure S14, Supporting Information). The XRD pattern of the TeNW @ CNT sheet reveals a dominating carbon peak around 26°, and small peaks at 38.5°, 40.5°, and 49.7° which correspond to a crystalline Te (Figure S15, Supporting Information). To utilize the TeNW @ CNT sheet as a Li‐metal anode substrate, a prelithiation process was carried out. A controlled amount of Li was electrodeposited onto TeNW @ CNT to uniformly distribute Li on the free‐standing sheet (Figure S16a, Supporting Information). The amount of Li deposited on the Li‐TeNW @ CNT was intentionally controlled to match the N/P ratio of 1, which is the same N/P ratio as the previously used Li_2_S‐cathode‐based anode‐free cells. Subsequently, the prelithiated Li‐TeNW @ CNT was assembled with a sulfur cathode at a loading of 3.2 mg cm^−2^, as shown in Figure S16b, Supporting Information. The total amount of TeNW applied to the Li‐TeNW @ CNT was 0.2 mg cm^−2^, which corresponds to 4 wt% compared to the weight of Li and S in the cell when converted to the mass of Li_2_S.

Prelithiated Li‐TeNW @ CNT combined with the sulfur cathode was cycled to evaluate the long‐term cycling performance. For the control cell, CNT without TeNW was vacuum filtered to make the free‐standing sheet and prelithiated to act as an anode (Li‐CNT). As the cathode started with the elemental sulfur, the specific capacities are calculated on the basis of the mass of sulfur. Figure S17, Supporting Information, demonstrates that the cell containing TeNW in the anode substrate exhibit a significant capacity retention of 57% (532 mAh g^−1^) after 200 cycles with a stable average Coulombic efficiency of 91.7%. In contrast, the control cell without TeNW in the system exhibits a rapid capacity decay after 50 cycles and shows a capacity retention of only 5% (42 mAh g^−1^) after 200 cycles with a poor average Coulombic efficiency of 85.4%. It is worth emphasizing that good cycling performance of these cells is achieved when Li and S are stoichiometrically balanced without excess lithium (N/P = 1). Therefore, it is shown that directly applying TeNW to the anode side can also effectively improve cycling performance with low Te content, as demonstrated in this study. The universal applicability of TeNW, not only as a coating material for separators but also as an anode substrate, can be a promising strategy to increase the lifespan of Li‐limited low N/P ratio Li–S batteries.

## 3 Conclusions

Various strategies incorporating TeNW into the system have been proven to significantly increase the lifespan of Li‐limited Li–S batteries. The nanosized high‐surface‐area property of TeNW has shown that it can effectively enhance the cell cycle life even with a low Te content. In addition, by isolating TeNW from electronically and ionically insulating sulfur or Li_2_S cathodes and incorporating them into the separator or anode side, the utilization of Te can be maximized, leading to an enhanced cyclability with a low TeNW content of only 4 wt% relative to the active material. TeNW plays a similar role in stabilizing the Li‐metal side as the existing Te mechanism by forming a good Li‐ion conductive SEI containing polytellurosulfides (Li_2_Te_*x*_S_*y*_) on Li surface. Therefore, it contributes to a dense and uniform deposition of Li metal, ultimately resulting in an innovative increase in cycle life, especially in Li‐limited systems. This work provides a rational design strategy to realize high‐energy density long cycle life anode‐free Li–S batteries.

## Conflict of Interest

The authors declare no conflict of interest.

## Supporting information

Supplementary Material

## Data Availability

The data that support the findings of this study are available from the corresponding author upon reasonable request.
