# Supplementary material for: Tellurium Nanowires for Lithium‐Metal Anode Stabilization in High‐Performance Anode‐Free Li–S Batteries
Source: Small Sci. 2023 Aug 22;3(10):2300088. doi: 10.1002/smsc.202300088 (PMC11935942; doi:10.1002/smsc.202300088)
Supplement: Supplementary file 1 — Supplementary Material [file SMSC-3-2300088-s001.pdf]

Supporting Information

Tellurium Nanowires for Lithium-Metal Anode Stabilization in High-Performance Anode-free Li–S Batteries

*Hyunki Sul, Jiarui He, and Arumugam Manthiram**

Experimental Section

Synthesis and preparation of Tellurium nanowire (TeNW)

0.178 g of Na_2TeO_3 and 2 g of polyvinylpyrrolidone (PVP) were dissolved in 50 mL of deionized water for 30 min. 3.3 mL of hydrazine monohydrate and 7.1 mL of aqueous ammonia solution were added and further stir-mixed for 2 h at room temperature. The solution was transferred into a polytetrafluoroethylene hydrothermal container and heated up to 180 ºC for 3 h at a ramp rate of 4 ºC min^{-1}. After cooling down the container to room temperature, the TeNW samples could be precipitated by adding an appropriate amount of acetone into the solution, washing with acetone several times, and drying in vacuum at 60 ºC.

Preparation of Li_2S cathodes

Commercial Li_2S (99.9 % metal basis, Sigma Aldrich), multi-walled carbon nanotubes (MWCNT, Nanostructured & Amorphous Materials Inc.), and Super-P were dry milled for 2 h with a long roll jar-milling system (US Stoneware 802 CVM) in a weight % ratio of 80: 10: 10. The resulting homogenously mixed composite was wet ball milled in a PTFE bottle with a mixture of 90 wt. % composite and 10 wt. % PEO/PVP binder in a 1,2-dimethoxyethane (DME), 1,4-dioxane, and acetonitrile solution media for 24 h. Yttria-stabilized zirconia (YSZ) grinding balls

were used to create a uniform slurry mixture. The slurry was blade casted onto an aluminum foil, and the electrode was dried inside an argon-filled glovebox under vacuum for 24 h to remove the solvent. Cathodes with a Li_2S loading of ~ 3 mg cm^{-2} and a Li_2S content of 72 wt. % were obtained. Commercial tellurium (Te, Sigma Aldrich) or TeNW was added to the slurry mixture in a weight ratio of 90: 10 and 95: 5 with Li_2S and blade casted onto an aluminum foil.

Preparation of Sulfur cathodes

Commercial S (99.5+%, Acros Organics) was melt-diffused into Ketjen Black (KB, 90 wt. % S content). The S/KB composite was mixed with MWCNT, super-P, and PEO/PVP in a wt. % ratio of 80: 5: 5: 10 in water. The slurry-cast sulfur cathode onto the aluminum foil consisted of 72 wt. % sulfur with a loading of 3.2 mg cm^{-2}.

Preparation of TeNW (& Te powder)-coated separator

TeNW was dispersed in water for 10 min. Pre-made 4 wt. % commercial Soteras CCS-V binder (Ashland) in an aqueous solution was added, and a CCS-B cross-linker was further added. The weight ratio between CCS-V and CCS-B was 10: 1, and the total TeNW: Binder weight % ratio was 95: 5. The final solution was kept stirring at room temperature for 30 min to obtain a homogeneous slurry. Finally, the slurry was blade casted onto one side of commercial polypropylene (PP) separator (Celgard 2500). Commercial Te powder was coated onto the separator with an analogous procedure. The Te powder-coated separator consisted of a Te: CNF: binder weight % ratio of 80: 10: 10. The coated separator was dried at 50 ºC for 5 min at atmospheric pressure.

Preparation of pre-lithiated Li-TeNW @ CNT and Li-CNT

TeNW and MWCNT were added into an ethanol : water (1:1 vol. %) solution in a weight % ratio of 10 : 90 and stir-mixed for 30 min. The obtained mixture was then intensively ultrasonicated for 30 min to form a uniform suspension. The entire solution was vacuum filtered to make the binder-free TeNW @ CNT sheet. For pre-lithiation, Li || TeNW @ CNT cells were assembled in CR-2032 type coin cells with Celgard 2500 separator and 1 M lithium bis(trifluoromethane sulfonyl)imide (LiTFSI) in 1,2-dimethoxyethane (DME) / 1,3-dioxolane (DOL) (1:1 vol. %) electrolyte. The amount of Li plated was controlled to match the N/P ratio of 1. Li-CNT was prepared in a similar method without the addition of TeNW.

Materials characterization

Morphological investigation was performed with a scanning electron microscope (FEI Quanta 650 SEM operated at 20 kV). X-ray photoelectron spectroscopy (XPS) was performed with a Kratos Analytical spectrometer with monochromatic Al Kα as a radiation source. Argon sputtering of the samples was conducted by exposing the sample surface to an Ar^+ ion beam for 10 min. A transmission electron microscope (JEOL 2010F field emission TEM) was used for obtaining high-magnification TEM images. X-ray diffraction (XRD) patterns were recorded with a Rigaku Miniflex 600 diffractometer at a scan rate of 0.5° min^{-1} with a step size of 0.02°. TGA data were collected with a thermogravimetric analyzer (Mettler-Toledo) in the temperature range of 50 – 800 ºC at a heating rate of 10 ºC min^{-1} under N_2 atmosphere.

Electrochemical cell assembly

The anode-free Ni || Li_2S full cells consisted of nickel foil as the anode substrate, separator, Li_2S-based cathodes, and 1 M LiTFSI + 0.25 M $LiNO_3$ in DME/DOL as an electrolyte. Pre-lithiated Li-CNT || S full cells were assembled with lithiated CNT sheet as the anode, separator, and S-based cathodes with the abovementioned electrolyte. All cells were assembled inside an argon-filled glovebox.

Electrochemical performance measurements

An Arbin battery cycler was used to conduct electrochemical cell cycling. For Li_2S-based anode-free cells, the cells were rested for 10 h at room temperature prior to the testing. Cells were activated at C/10 for 3 cycles between 1.8 V to 3.5 V, and the following cycles were conducted at C/5 (1.8 V – 3.0 V). 1C corresponds to a current density of 1,166 mA g^{-1} and the specific capacities were calculated based on the total Li_2S mass. For the shuttle current test, cells were initially charged to 3.5 V and then discharged to 2.35 V. After reaching 2.35 V, the voltage was maintained to record the steady-state current response resulting from the polysulfide shuttling. For S-based full cells, the cells were rested for 4 h and cycled at C/5 without activation (1C = 1,675 mA g^{-1}). Electrochemical impedance spectroscopy (EIS) measurements were performed with a Biologic VMP potentiostat in the frequency range of 1 MHz to 0.1 kHz.

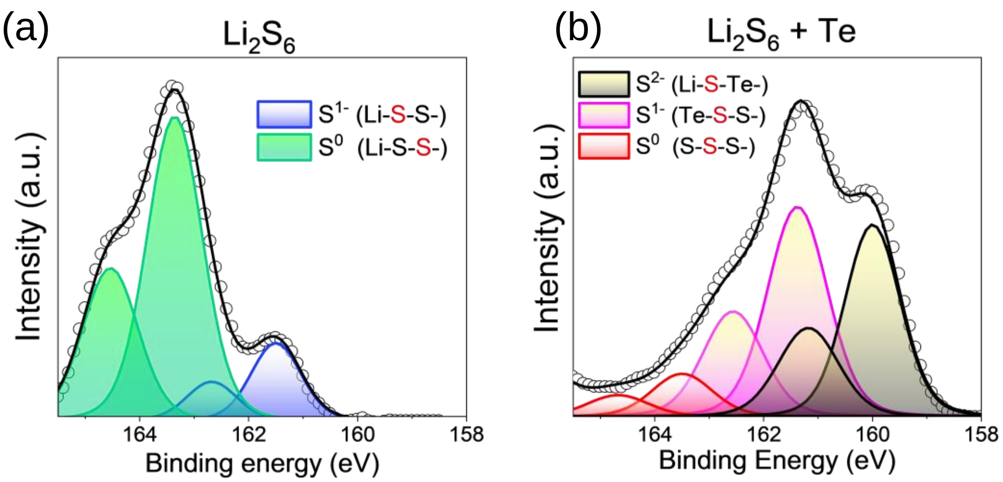

Figure S1. S 2p XPS data of Li_2S_6 and Li_2S_6 + Te at the surface. Through the reaction between Li_2S_6 and Te, S got reduced from S^0 to S^{1-} and S^{2-}.

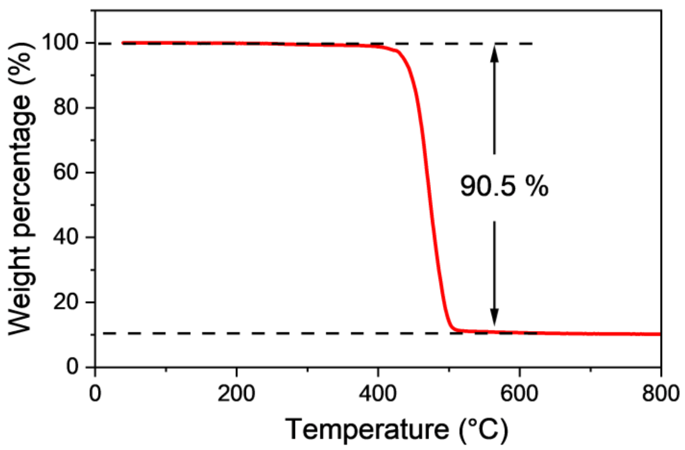

Figure S2. TGA curves in N_2 atmosphere of synthesized TeNW. TeNW yields a Te content of 90.5 and a carbon content of 9.5 wt. %.

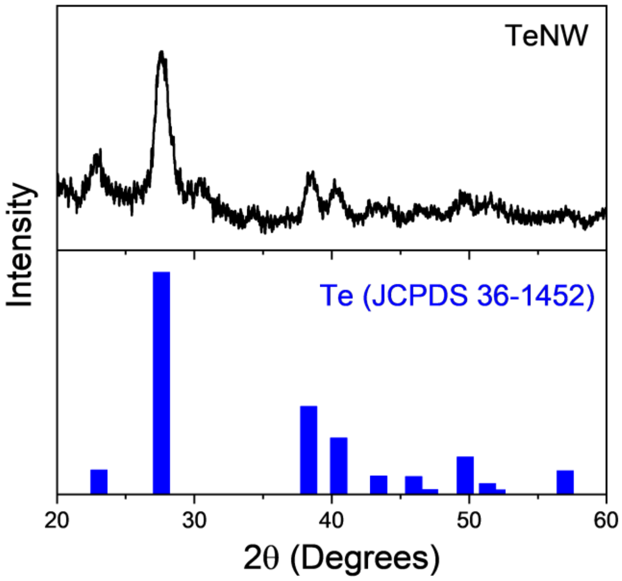

Figure S3. XRD pattern of synthesized TeNW and the reference peaks of Te.

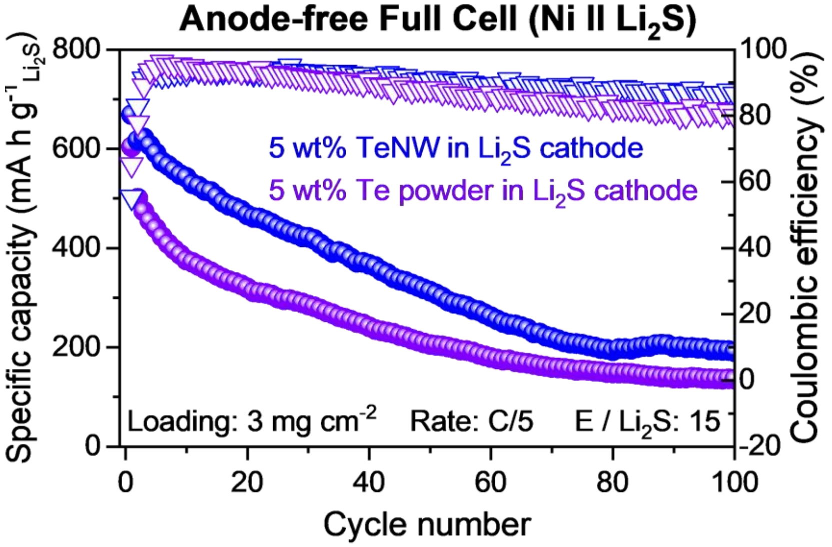

Figure S4. Long-term cycling performance of anode-free Ni || Li_2S full cells with 5 wt. % TeNW and commercial Te powder applied at the cathode.

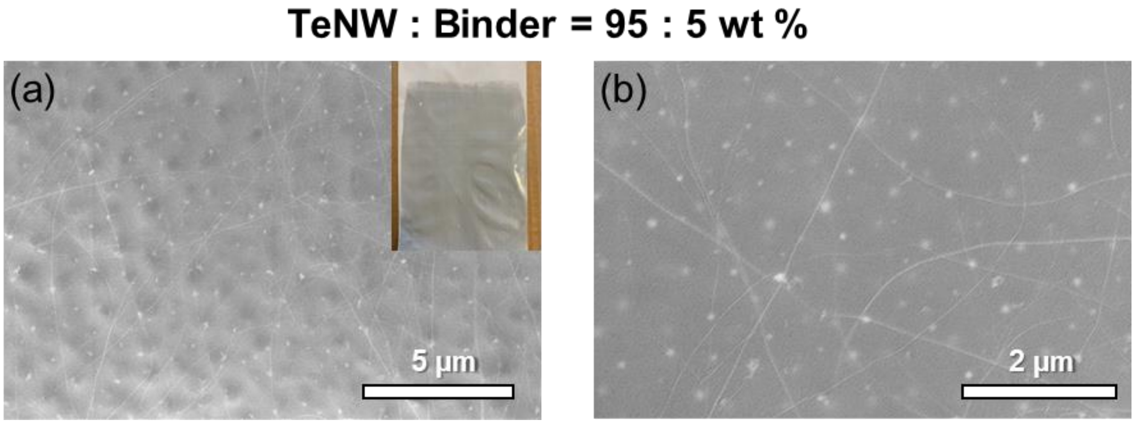

Figure S5. (a) SEM image of TeNW-coated separator. Inset: Digital image of the wide-region TeNW coating onto the separator. (b) Magnified image of TeNW on the separator.

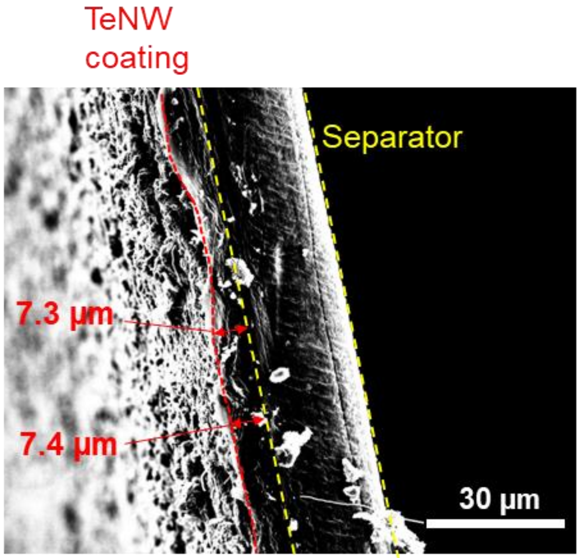

Figure S6. Cross-sectional image of TeNW-coated separator.

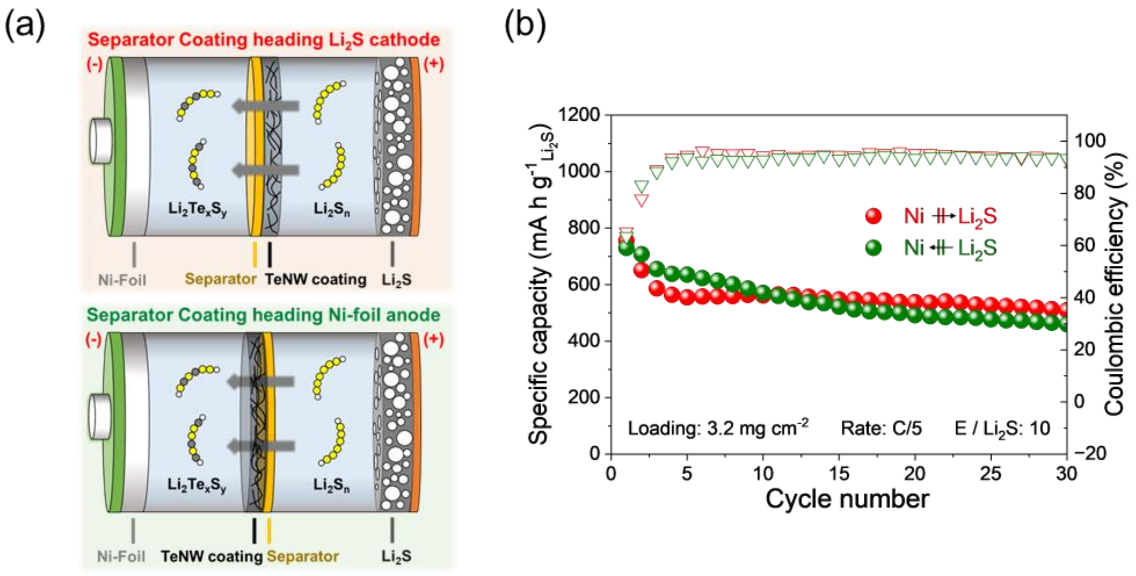

Figure S7. (a) Schematic of the two different TeNW-coated separator orientations: coated-side facing the Li_2S cathode and coated-side facing the Ni-foil anode. (b) Cell cycling performance of TeNW-coated separator facing the cathode side (red) and the anode side (green).

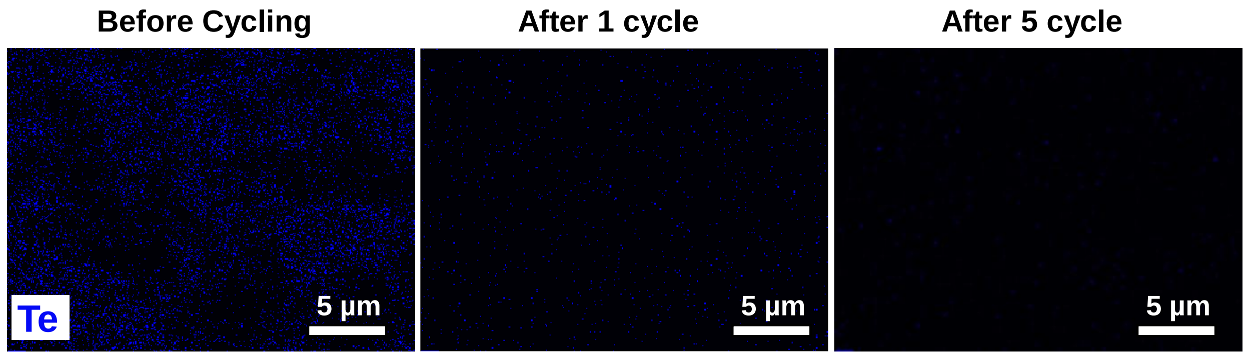

Figure S8. Elemental mapping of Te on the TeNW-coated separator before cycling, after 1 cycle, and after 5 cycles. The cycled separators were examined at the discharged state.

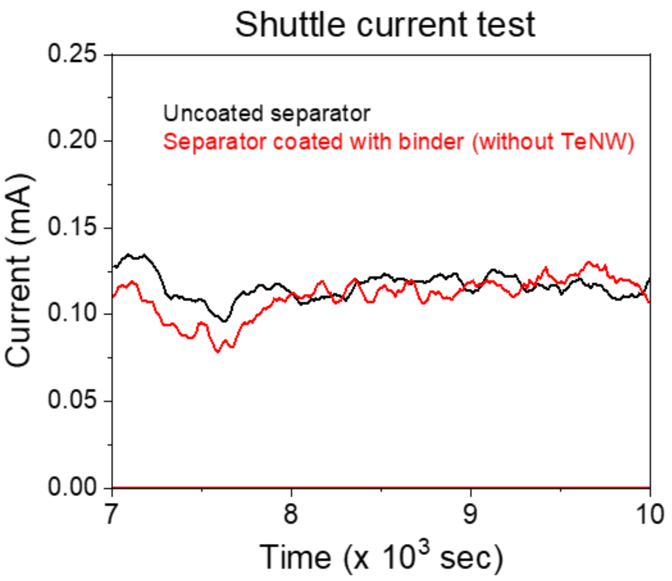

Figure S9. Shuttle current test for the uncoated separator (black) cell and for the cell in which the separator was coated only with the binder without TeNW (red). The similar shuttle current shows that the separator coating without TeNW has negligible impact on suppressing polysulfide shuttle.

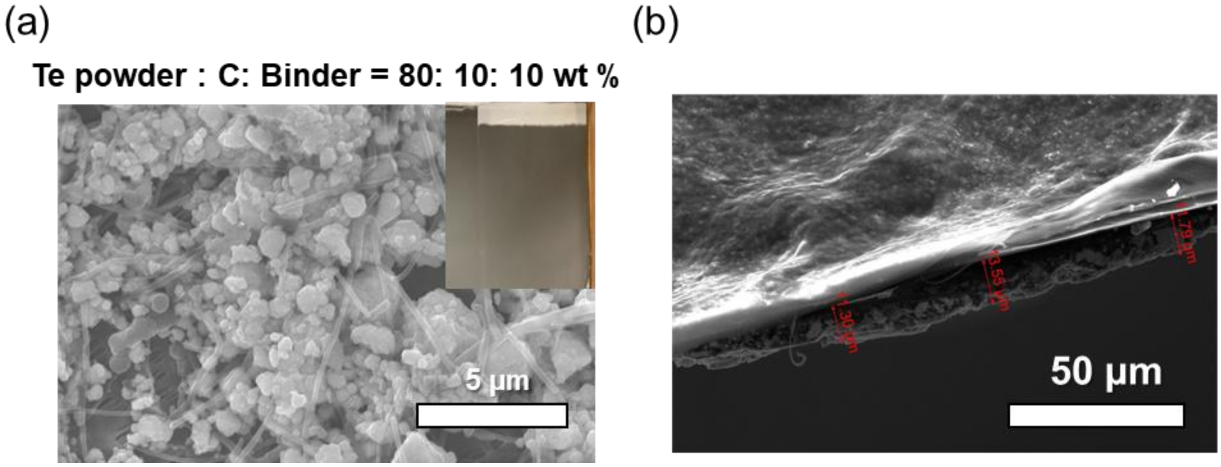

Figure S10. (a) SEM image of commercial Te powder-coated separator. Inset: Digital image of the wide-region Te powder coating onto the separator. (b) Cross-sectional image of commercial Te powder-coated separator.

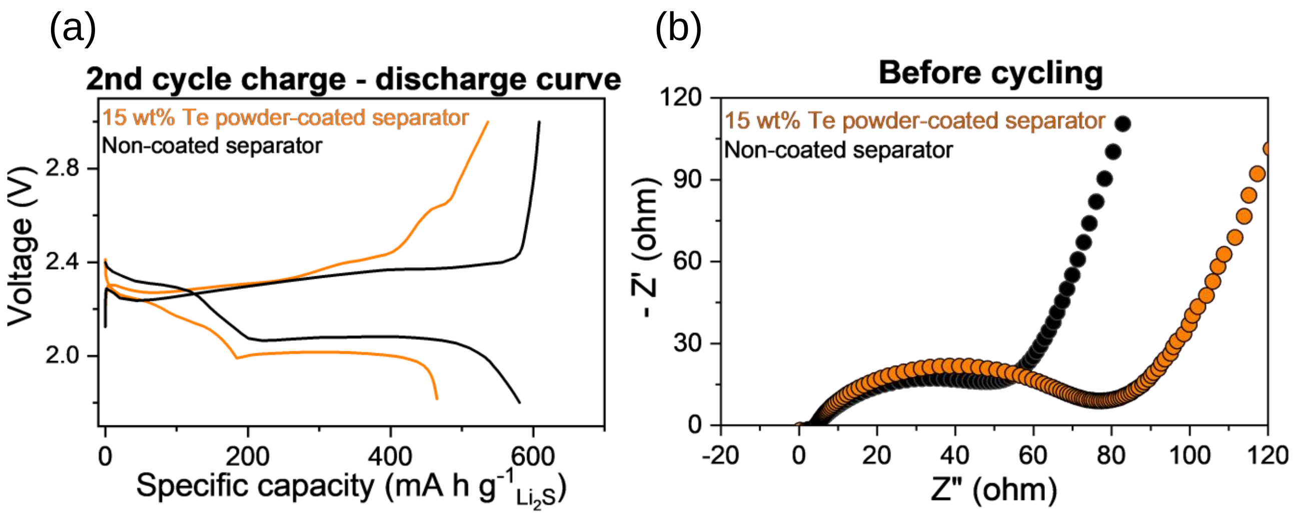

Figure S11. (a) 2nd cycle charge-discharge curves, and (b) Nyquist plots before cycling of 15 wt. % commercial Te powder-coated separator cell and uncoated separator cell.

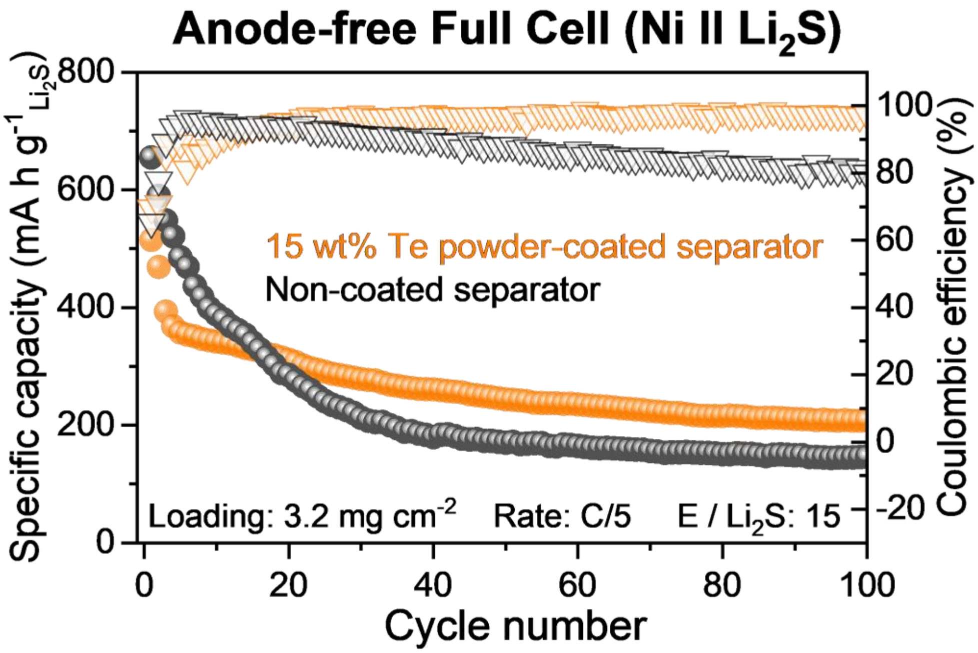

Figure S12. Long-term cycling performance of anode-free Ni || Li_2S full cells with 15 wt. % commercial Te powder-coated separator cell and uncoated separator cell.

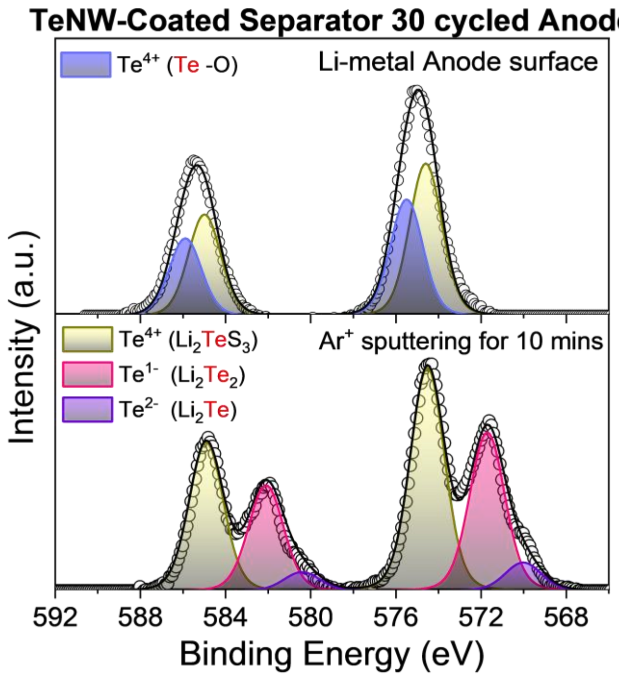

Figure S13. Te 3d XPS spectra of Li-metal surface after 30 cycles of TeNW-coated separator cell before and after Ar^{+} sputtering for 10 min. The reaction mechanism and formation of polytellurosulfide species at Li-metal surface can be written as follows:

$$Te^0 + Li_2S_n \rightarrow Li_2TeS_{3+x}\ (Te^{4+})$$

$$Li_2TeS_{3+x}\ (Te^{4+}) + \ 2\ Li^0 \rightarrow Li_2TeS_3\ (Te^{4+}) + Li_2S_x$$

$$2\ Li_2TeS_3\ (Te^{4+}) + 10\ Li^0 \rightarrow Li_2Te_2\ (Te^{1-}) + 6\ Li_2S$$

$$Li_2Te_2\ (Te^{1-}) + 2\ Li^0 \rightarrow 2\ Li_2Te\ (Te^{2-})$$

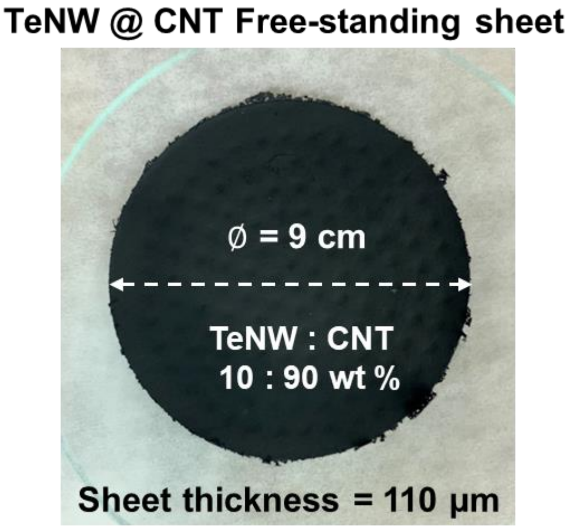

Figure S14. Digital image of vacuum-filtered TeNW @ CNT free-standing sheet.

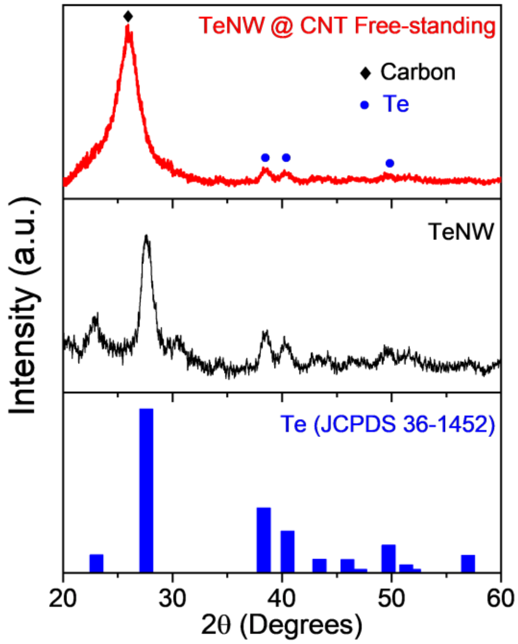

Figure S15. XRD patterns of TeNW @ CNT free-standing sheet and TeNW only, with the reference peaks of Te.

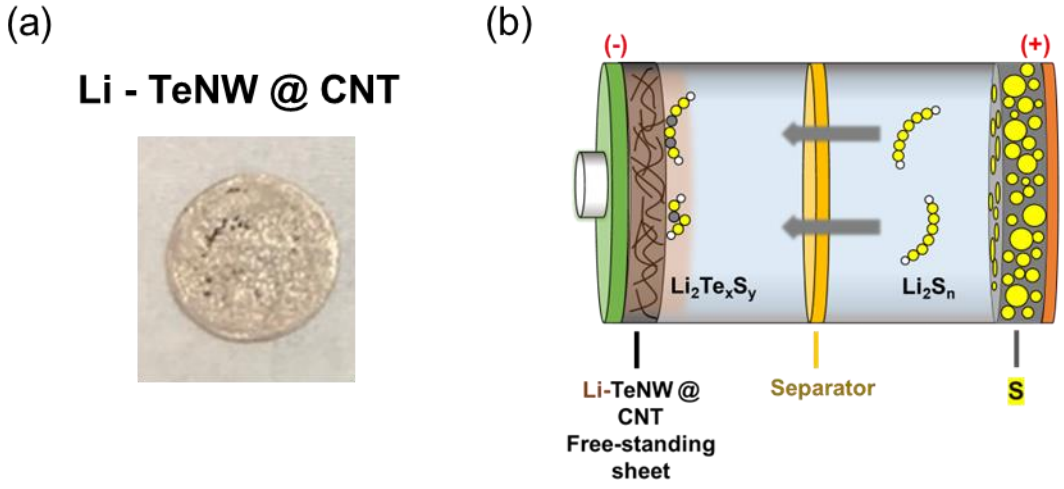

Figure S16. (a) Digital image of pre-lithiated Li-TeNW @ CNT. (b) Schematic of the Li-TeNW @ CNT || S full cell configuration. The N/P ratio was controlled to 1.

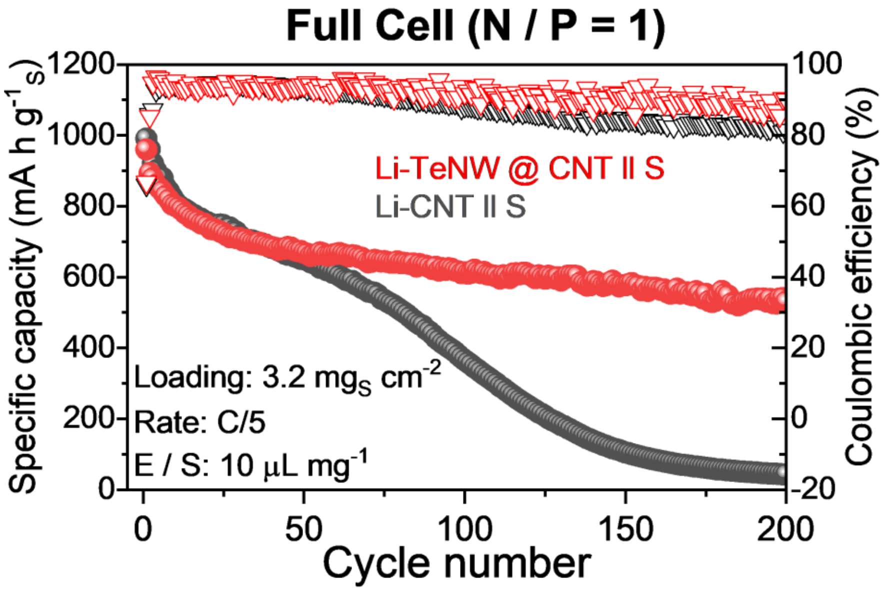

Figure S17. Long-term cycling performance of Li-TeNW @ CNT || S and Li-CNT || S full cells.

Table S1. Weight fraction contributions of each cell component in Li-S cells with practical parameters with different amounts of Te addition to the cathode compared to Li_2S

Li_2S Loading: 5 mg cm^{-2}, **Area:** 24 cm^2, **E/Li_2S:** 5 µl mg^{-1}

Addition of Te compared to Li_2S (wt. %)	Mass contribution (wt. %)							
	Cathode				Al-current collector (Cathode)	Separator	Ni-current collector (Anode)	Electrolyte
	Sulfur	Carbon	Binder	Te				
20	11.48	2.87	1.59	2.30	3.87	2.30	9.57	66.02
10	11.62	2.90	1.61	1.16	3.92	2.32	9.68	66.79
5	11.68	2.92	1.62	0.58	3.94	2.34	9.74	67.18
0	11.75	2.94	1.63	0	3.96	2.35	9.79	67.58
